# Comment On: Vonoprazan on the Eradication of *Helicobacter pylori* Infection

**DOI:** 10.5152/tjg.2023.23130

**Published:** 2023-04-01

**Authors:** Tolga Düzenli, Hüseyin Köseoğlu, Muhammed Kaya, Mesut Sezikli

**Affiliations:** 1 Department of Gastroenterology, Hitit University Faculty of Medicine, Çorum, Turkey

Dear editor,

We read the article “Vonoprazan on the Eradication of *Helicobacter pylori* Infection” with interest.^1^ The authors investigated the efficacy and safety of vonoprazan in the eradication of *Helicobacter pylori* (*H. pylori*) and concluded that both vonoprazan triple therapy and vonoprazan quadruple therapy regimens could increase the eradication rate of *H. pylori*. We found these findings important and would like to thank the authors for their contribution, yet we would also like to draw the readers’ attention to some methodological issues. First, the treatment regimens included furozalidone in the study. The authors pointed out the problem of resistance of *H. pylori* in Western literature. However, furozalidone is not a commonly used drug in Europe and Turkey in the first line. Furthermore, furozalidone is a drug of diminishing interest in Maastricht VI/Florence consensus report.^2^ Also, other bismuth-containing or concomitant therapies have good eradication rates for *H. pylori*. Why did the authors choose furozalidone as first-line treatment? Could it be that it was cheaper and easily available in China? Second, it was not emphasized in the paper whether the treatments were given as first line or after treatment. It seems likely that it was used in the treatment of naive patients, but it would be better if that was highlighted. Third, esomeprazole was used with a dose of 2 × 20 mg in the traditional quadruple therapy group in the present study. In the literature, it is more common to use esomeprazole with a dose of 2 × 40 mg or 4 × 20 mg for the eradication of *H. pylori*.^3,4^ Fourth, 14C urea breath test was used for the diagnosis of *H. pylori*. Would it be better to make a baseline upper gastrointestinal system endoscopy to see if there was peptic ulcer or another complication of *H. pylori*? If such data existed, the contribution of the study to clinical practice would have been strengthened.

Low-cost, non-invasive, simple, and effective primary regimens are still needed for the treatment of *H. pylori*, and options vary by region and treatment regimen. We believe that the findings of Huang and Lin^1^ will lead to further research concerning the treatment of *H. pylori*. On the other hand, further large-scale, prospective, randomized, and well-designed studies are needed to validate these findings in other countries or regions.
